# Disrupting Mechanisms that Regulate Genomic Repeat Elements to Combat Cancer and Drug Resistance

**DOI:** 10.3389/fcell.2022.826461

**Published:** 2022-05-04

**Authors:** Chames Kermi, Lena Lau, Azar Asadi Shahmirzadi, Marie Classon

**Affiliations:** Pfizer Center for Therapeutic Innovation., San Francisco, CA, United States

**Keywords:** transposable elements, drug resistance, epigenetic regulation, restriction factors, R-loops, nucleic acid sensing, innate and adaptive immunity

## Abstract

Despite advancements in understanding cancer pathogenesis and the development of many effective therapeutic agents, resistance to drug treatment remains a widespread challenge that substantially limits curative outcomes. The historical focus on genetic evolution under drug “pressure” as a key driver of resistance has uncovered numerous mechanisms of therapeutic value, especially with respect to acquired resistance. However, recent discoveries have also revealed a potential role for an ancient evolutionary balance between endogenous “viral” elements in the human genome and diverse factors involved in their restriction in tumor evolution and drug resistance. It has long been appreciated that the stability of genomic repeats such as telomeres and centromeres affect tumor fitness, but recent findings suggest that de-regulation of other repetitive genome elements, including retrotransposons, might also be exploited as cancer therapy. This review aims to present an overview of these recent findings.

## Introduction

**FIGURE 1:**
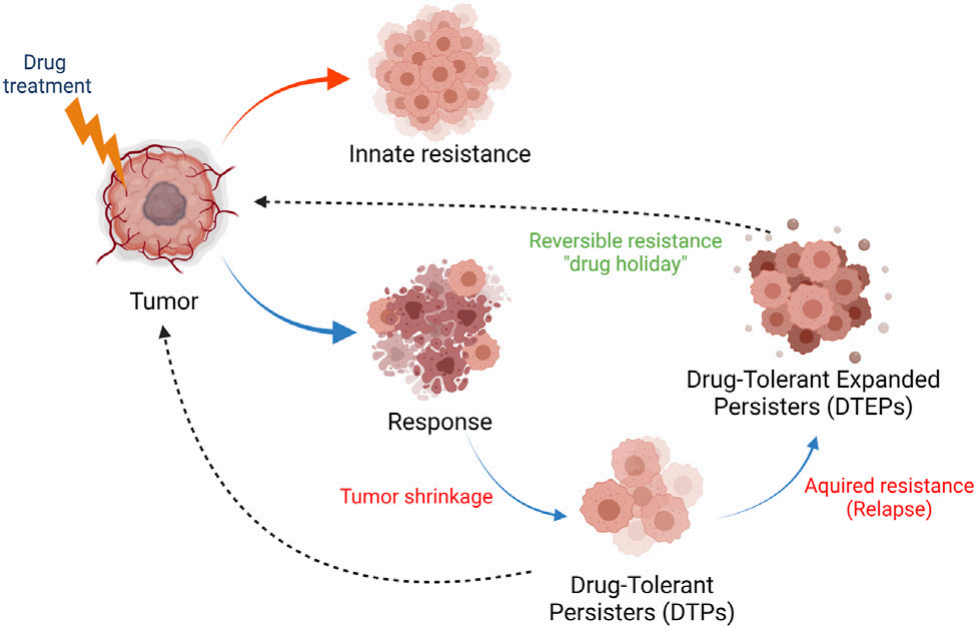
Innate and acquired drug resistance in cancer. Tumors can display innate resistance to therapy (red arrow), the causes and origins of which are not completely understood. Tumors can also develop acquired therapy resistance (blue arrows). Upon exposure to therapy and response, a fraction of tumor cells survive the initial lethal drug exposure–drug-tolerant persister (DTPs) cells. This dormant state, which can be reversible, contributes to therapy relapse or the establishment of drug-tolerant expanded persister cells (DTEPs) that harbor many genetic and epigenetic changes developed in order to adapt to the drug exposure. It should be noted that rare pre-existing resistance mutations have also been found in human tumors. The DTEP state can, following a “drug holiday,” return to a drug-responsive tumor state (black, dotted arrow), a phenomenon reported in tissue culture and in patients. Many mutational, non-reversible, resistance mechanisms have also been described in tumors from patients that have relapsed on therapy.

Despite many recent advances in the treatment of cancer, such as rationally-targeted agents (based on the concept of oncogene addiction) and immune-oncology (IO) therapies (Seebacher et al., 2019; Zhang and Zhang 2020), drug resistance has nonetheless remained an obstacle to achieving long-term cancer remissions or cures (De Conti et al., 2021). Resistance to drug treatment can be broadly categorized as innate and acquired resistance (Figure 1). Innate resistance is defined by the absence of a measurable clinical response to drug treatment, while acquired resistance is defined as disease progression following an initial response to treatment. With respect to IO therapy, innate resistance has been attributed to the fact that many tumors seem to be immunologically “cold” and are described as “immune deserts,” but mechanisms for innate resistance generally remain poorly defined in the context of targeted agents and chemotherapies (Graudens et al., 2006).

Many mechanisms that underlie acquired resistance to chemotherapeutic or targeted agents have been described (Vasan et al., 2019). Most genetic or epigenetic changes in relapsed tumors originate in a subpopulation of cells that survive the initial lethal drug exposure and serve as founders for therapeutic relapse - drug-tolerant persister cells (DTPs) (Roesch et al., 2010; Sharma et al., 2010; Somasundaram et al., 2012; Ramirez et al., 2016; Somasundaram et al., 2016; De Conti et al., 2021). The development of acquired resistance, therefore, largely reflects pre-existing intrinsic tumor heterogeneity defined by genetic, metabolic, and/or epigenetic states; the latter two can be reversible and stochastic (De Conti et al., 2021). Most of the existing data that define genetically determined acquired resistance mechanisms have been derived from genomic sequencing of bulk tumor material from relapsed patients. Due to the lack of extensive clinical data sets from patient-matched biopsies collected pre- and post-treatment, our understanding of DTPs from clinical samples is limited. However, the development of DTP cell culture models and single-cell sequencing technologies have enabled a better understanding of genetic mechanisms that underlie tumor heterogeneity and drug response (Sharma et al., 2010; De Conti et al., 2021; Kashima et al., 2021). The non-genetic, reversible, and regulatory mechanisms that define drug resistance are not well-defined, and a better understanding of these mechanisms will provide new insights into the underlying biology that shape drug responses.

Acquired genetic resistance mechanisms that emerge during IO therapies have been identified in relapsed patients, and include tumor mutations in the Interferon-gamma (IFN-γ), Janus kinase (JAK)-signal transducer and activator of transcription (STAT) pathways, as well as major histocompatibility complex (MHC) components (Zaretsky et al., 2016; Sucker et al., 2017; Christopher et al., 2018). The existence of “DTPs” in the IO treatment setting has also been recently described, but there is a limited understanding of the mechanisms that provide a survival benefit for these cancer cells (Sehgal and Barbie 2021; Sehgal et al., 2021).

**FIGURE 2:**
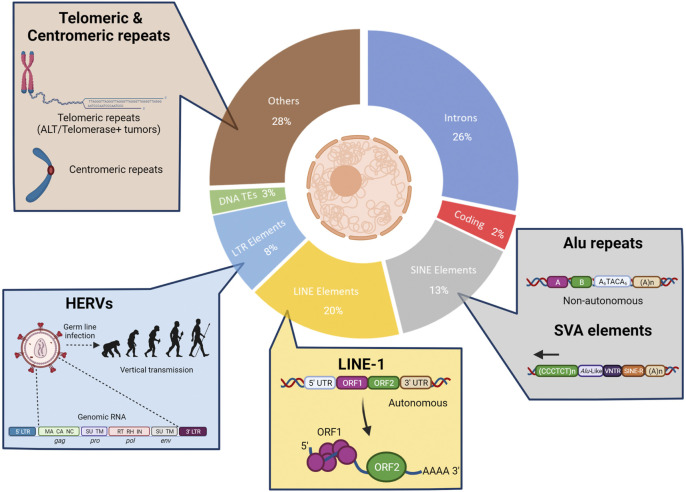
The under-explored parts of the human genome. Most cancer resistance mechanism studies have focused on the non-repetitive protein-coding parts of the human genome (2%, red). However, a large part of the human genome consists of non-coding sequences that comprise the intergenic DNA, centromeric and telomeric repeats (light brown) as well as integrated viruses and transposable elements (TEs) that have been replicated in the human genome during evolution (nearly 50% of the genome). The non-LTR TEs represent almost one-third of the human genome and include non-autonomous Short Interspersed Nuclear Elements (SINEs; grey) and autonomous Long Interspersed Nuclear Elements (LINEs; yellow). LTR TEs include human endogenous retroviruses (HERVs, light blue). There are also remnants of DNA transposons (green).

The current knowledge of mechanisms that contribute to the evolution of tumors and drug resistance has been derived almost exclusively from the evaluation of the 2 percent of the human genome that represents non-repeat protein-coding regions (Figure 2). As mentioned above, extensive sequencing efforts centered around these genomic regions have resulted in the identification of a multitude of genetic alterations that contribute to tumorigenesis, treatment response, and resistance. However, the vast repetitive regions of the genome (Lander et al., 2001) remain under-explored in the context of tumor development and drug resistance. Initially considered “junk,” a biological role for repetitive elements was first suggested by Barbara McClintock almost a century ago. Her landmark studies identified mobile sequences that are able to change their position within a genome and affect biological states. Investigations over the subsequent decades have revealed important roles for these elements across evolution, development, disease progression, and adaptation to fluctuating environments, including drug treatment (McClintock 1929; Kazazian et al., 1988; Dombroski et al., 1991; Moran et al., 1996; Huang et al., 2010; Payer and Burns 2019). Recent improvements in sequencing technologies and bioinformatic analyses have enabled a rapidly emerging understanding of genome repeat biology (Mantere et al., 2019; O'Neill et al., 2020). Activation of transposable elements (TEs) can result in genomic alterations (mutations and chromosomal re-arrangements), modifications to the three-dimensional organization of the genome, transcriptional changes, and generation of nucleic acid species (NAS) that may be detected by innate immune sensing machinery (Chen et al., 2021; Klein and O'Neill 2018). Despite the potential physiological benefits of TE integration and propagation throughout the genome, the consequences of TE activation can also be detrimental; therefore, many “counter-balancing” mechanisms have co-evolved with TE integration to keep these potential negative effects in check.

In this review, rather than presenting a comprehensive overview of cancer drug resistance and TE regulatory mechanisms in various biological contexts, we aim to provide a perspective on the role of these ancient integrated viral sequences and their restriction factors in the context of tumor evolution and drug resistance. Several recent discoveries have suggested a paradigm in which differential activation of genomic TEs, such as endogenous retroviruses (ERVs) and Short and Long INterspersed Elements (SINEs and LINEs), can affect tumor progression and cancer therapy responses, impacting cancer cell fitness as well as innate and adaptive immune responses. Here, we summarize various reported mechanisms involved in the regulation of genomic repeat elements, discuss how the de-repression of such elements may influence cancer drug responses, and provide insights into novel therapeutic strategies intended to specifically target tumor cells as well as overcome drug resistance.

## The Under-Explored Parts of the Human Genome

The conservation of TEs and other repeats in the human genome suggests that they have conferred a selective advantage during the evolution and survival of the species (Cordaux and Batzer 2009; Feschotte and Gilbert 2012). As depicted in Figure 2, a large fraction of the human genome is comprised of repetitive sequences. Many of these sequences can contribute to genetic structural variation in the human population as well as the development of a variety of maladies, including cancer, auto-immunity, and neurodegenerative diseases (Payer and Burns 2019).

There are several types of repetitive sequences in the human genome, including telomeric and centromeric repeats, DNA transposons, and multiple classes of TEs. These elements and their potential role(s) in cancer are described in some detail below.

### Telomeric and Centromeric Repeats

Work from McClintock and Muller first showed that telomeres, repetitive sequences at the ends of eukaryotic chromosomes, have important biological functions in maize and *drosophila* (McClintock 1938, 1941; Muller 1938). In the almost 100 years since these discoveries, many studies have shown that mechanisms maintaining telomere repeat length are crucial for the stability of genomes and the preservation of human health and longevity (Lu et al., 2021). Human telomeric regions consist of tandem TTAGGG repeats that extend several kilobases at the end of chromosomes and terminate in a 3′ single-stranded overhang (50–400 nucleotides). Telomeric chromosome ends structurally resemble double-strand breaks (DSB), and many mechanisms have evolved to protect chromosomes from telomere erosion or the initiation of a DNA damage response (DDR) at telomeres (Palm and de Lange 2008; Fumagalli et al., 2012; Okamoto et al., 2013; Schmutz and de Lange 2016). For example, long-term telomere maintenance during development and in germ cells is ensured by the activity of telomerase, an enzyme that consists of protein and RNA components that add TTAGGG sequences to chromosomal ends during replication. Telomerase activity is relatively low in somatic, differentiated cells but can become amplified in tumor cells to prevent telomere erosion during rapid proliferation. However, there are also “telomerase-negative” tumor cells that use homologous recombination pathways to maintain telomere length in a process called Alternative Lengthening of Telomeres (ALT) (Bryan et al., 1997; Dilley and Greenberg 2015). De-regulation of the mechanisms that maintain the integrity of telomeric repeats has long been recognized as a vulnerability that can be exploited to improve therapeutic responses in cancer.

Centromeric repeats are highly specialized chromatin domains that connect chromosomes to the mitotic spindle and play an important role in chromosome segregation. Human centromeres are comprised of tandem arrays of alpha-satellite DNA repeats that can extend several mega-bases (Goldberg et al., 1996; Alkan et al., 2007). The repeats in peri-centromeric regions (flanking centromeres) are more unstructured and heterogeneous. Alpha-satellite DNA is often monomeric within these regions and is interspersed with other repetitive elements, including LINEs and SINEs (Klein and O'Neill 2018). In addition to alpha-satellite repeats, other types of satellite repeats exist in the human genome, such as the 5-bp satellite II and satellite III repeats (HSATII and HSATIII) (Garrido-Ramos 2017). In some cancers, the transcription of such satellite-rich pericentromeric regions can result in the generation of non-coding RNA (ncRNA) and RNA–DNA hybrid accumulation that can contribute to the instability of these regions. Increased expression and propagation of HSATII repeats have been observed in many epithelial solid tumors (Bersani et al., 2015). Repressive DNA methylation has been implicated in centromeric array regulation and stability, a mechanism that can be altered in tumors. The role that centromere and telomere stability plays in the emergence of drug resistance is currently not well understood.

### Repetitive Transposable Elements

The TEs in the human genome can be subdivided into DNA transposons and retrotransposons. Classical DNA transposons, originally discovered by Barbara McClintock in maize, transpose via a “cut-and-paste” mechanism in which they are excised out of the genome and re-inserted elsewhere. Although prevalent in some species, DNA transposons are most likely no longer active in humans, but their remnants still constitute approximately 3 percent of the human genome. Retrotransposon DNA generates RNA intermediates before being reverse-transcribed into DNA and inserted into the genome as a new copy. This class of transposons can be further divided into long-terminal repeat (LTR) and non-LTR retrotransposons. Most human retrotransposons have also been rendered inactive by mutations and no longer undergo autonomous retro-transposition in the genome. However, a fraction remains active, and mis-regulated expression of such retrotransposons can lead to damage in the genome, in part by generating DSBs. The only human autonomous retrotransposons are LINE-1 elements; a small subset of which encode all the components needed for retrotransposition. These LINE-1-encoded proteins can also promote retrotransposition of the nonautonomous SINE elements. These TEs, as well as the potential consequences of their deregulated expression in cancers, are described in more detail below.

Human LTR retrotransposons occupy 5-8 percent of the human genome and include families of human endogenous retroviruses (HERVs) that were incorporated as retroviruses, amplified, and domesticated throughout recent evolution. Full-length HERVs are comprised of LTRs flanking an open reading frame (ORF) that includes sequences encoding gag, pol, and env proteins. Most of the more intact HERV families have been recently incorporated into the human genome, and few, if any, retain the ability to generate viral particles or retrotranspose (Marchi et al., 2014; Naveira et al., 2014). However, HERVs can still play active roles in the human genome; their LTRs can affect transcription, generate novel fusion transcripts, as well as affect 3D genome organization (Thompson et al., 2016). LTR-mediated transcription can also generate NAS, which can be detected by innate immune sensors, thereby prompting a cell-intrinsic antiviral response. It has also been suggested that such a response can be further amplified by LTRs influencing interferon (IFN)-responsive genes directly (Roulois et al., 2015; Chiappinelli et al., 2016; Canadas et al., 2018). Through these mechanisms, deregulated HERVs may contribute to innate and adaptive immune responses towards tumors.

The expansion of the evolutionarily oldest TE class, LINE-1 elements, has been extensive; this group makes up approximately 17 percent of the human genome (approximately 500,000 copies). A large fraction of these elements are degenerate remnants, and only 7000 human LINE-1 elements are still maintained as full-length elements. The approximately 6 Kilobase (kb) full-length LINE-1 elements contain a 5′ untranslated region (UTR) that functions as a sense promoter, a 3’ UTR that terminates in a poly A signal, and three open reading frames, ORF1, ORF2, and ORF0, the latter being transcribed from a weaker promoter on the antisense strand (Beck et al., 2011; Denli et al., 2015). Human LINE-1 (L1)-ORF1 encodes a protein with RNA binding and chaperone capabilities, and L1-ORF2 encodes for a protein with both endonuclease and reverse transcriptase capabilities, both of which are required for retro-transposition. An estimated 100 human LINE-1 copies retain the capacity to retrotranspose and can contribute to genomic diversity in populations. Recent discoveries have suggested that deregulated LINE-1 expression can exhibit tumor-suppressive effects in tumor evolution as well as play a role in the potentiation of cancer therapy responses (Guler et al., 2017; Gu et al., 2021; Zhao et al., 2021).

The non-LTR SINE elements make up approximately 12 percent of the human genome. These non-autonomous elements are derived from transfer RNAs (tRNAs) and ribosomal RNA (rRNAs), do not encode for any proteins, and are dependent on the LINE-1 “machinery” for their propagation (Richardson et al., 2015). The largest class of human SINE elements is derived from signal recognition particle (7SL) RNA and have been named Alu elements, as they harbor an Alu restriction site in their sequences. Alu elements are approximately 300 base pairs (bp) in length and can be sub-classified into repeated inverted, non-inverted, and single elements (Richardson et al., 2015). In the context of cancer, it is noteworthy that de-regulated expression of inverted Alu elements has been shown to contribute to the induction of NAS and IFN signaling (Roulois et al., 2015; Mehdipour et al., 2020). Another class of SINE elements, the SINE-VNTR-Alu (SVA) repeats, are evolutionarily young retrotransposons comprising 0.2 percent of the human genome (Hancks and Kazazian 2010). Like LINE-1 and Alu elements, this class of active TEs can affect host cells in a variety of ways, including generation of mutations, exon shuffling, altered splicing, and production of NAS, all of which may contribute to various disease states and cancer drug responses.

It is indisputable that TEs have had a dynamic effect on genome development throughout evolution, and discoveries in recent years have unraveled roles for these elements in the development of many diseases including cancer, as well as in tumor therapy responses.

## Epi-Transcriptomic and Post-Transcriptional Lines of Defense

As mentioned previously, TEs can have positive and negative effects on their host, and the expansion of the repeat genome has therefore co-evolved with a myriad of defense mechanisms that counteract their potentially deleterious effects. While studies in recent years have improved our understanding of the role of TEs in cancer, we have only scratched the surface in our understanding of what role their regulation plays in the evolution of tumors and drug resistance.

### Potential consequences of de-regulated repeats–why is a defense needed?

**FIGURE 3:**
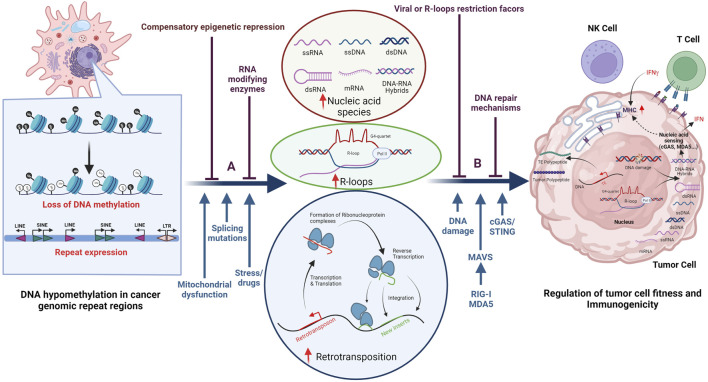
Establishment of an evolutionarily conserved truce contributes to cancer cell survival. *Cancer* cells often display DNA hypomethylation (open black circles) in the repetitive parts of the human genome, as displayed in the left part of the figure. De-repression of these regions can result in the accumulation of various nucleic acid species (NAS), R-loop formation, as well as changes in gene expression and/or DNA damage as illustrated in the middle part of the figure. The consequences for the tumor cell are illustrated in the right part of the figure and include induction of DDR as well as activation of the innate and adaptive immune system. Illustrated above the horizontal blue arrows are epi-transcriptomic **(A)** and post-transcriptional **(B)** mechanisms that have been proposed to counteract the negative effects that de-regulation of repeat regions has on cancer cell survival. Notably, some of these factors belong to both epi-transcriptomic and post-transcriptional categories. Indicated below the horizontal blue arrows are factors that contribute to decreased cell fitness as well as viral sensing mechanisms. Besides de-regulated repeat expression, other factors such as endogenous replication stress, DNA damage, splicing mutations, and some mitochondrial dysfunctions can all lead to increased NAS in the cytosol.

Humans harbor two types of immune defenses, adaptive and innate immunity, both of which can be activated by endogenous and exogenous viruses. It has long been appreciated that exogenous viruses can be detected by different classes of pattern recognition receptors (PRRs) to produce an IFN response. These viral sensing receptors include Toll-like receptors (TLRs) and retinoic acid-induced gene-I (RIG-I)-like receptors (RLRs), the latter including RIG-I and melanoma differentiation-associated protein 5 (MDA5), which recognize double-stranded RNA (dsRNA) species. In addition, there is cyclic GMP-AMP synthase (cGAS)/stimulator of IFN genes (STING) and absent in melanoma-2 (AIM2), which recognize double-stranded DNA (dsDNA), single-stranded DNA (ssDNA) as well as DNA/RNA hybrids (Schlee and Hartmann 2016). In cancers, it has been shown that de-regulation of TE expression and the subsequent generation of various NAS can activate an innate immune response characterized by type I IFN production (Fukuda et al., 2021; Sheng et al., 2018; Zhao et al., 2021). This response has been termed “viral mimicry” and can result in decreased tumor cell fitness and/or increased tumor cell immunogenicity (Cuellar et al., 2017; Rooney et al., 2015; Roulois et al., 2015). In addition, TE-derived peptides can give rise to “neo-antigens,” whose presentation by MHC proteins on the surface of tumor cells can trigger an adaptive anti-tumor immune response (Kong et al., 2019; Laumont et al., 2018), as depicted in Figure 3. Innate immune cells, such as natural killer (NK) cells, whose ligands can be activated in damaged or virally infected cells, also contribute to tumor cell killing (Pech et al., 2019).

De-regulation of TEs and other repeats can result in DDR activation, which may also affect the fitness and immunogenicity of tumor cells. Repetitive elements, including telomeres, LINEs, and DNA transposons (Zeng et al., 2021), are prone to form secondary DNA structures during transcription. These include t-loops and G-quadruplexes (G4), which can stall replication machinery, increase replication stress, and induce DNA damage. Such transcription/replication conflicts can also result in the formation of R-loops (Santos-Pereira and Aguilera 2015), a three-stranded structure containing a DNA/RNA hybrid and a displaced ssDNA strand. The DNA/RNA hybrid can be excised and exported into the cytoplasm, where it can activate the cGAS/STING pathway (Coquel et al., 2018; Crossley et al., 2019), while the remaining ssDNA may induce DDR. DNA damage in tumor cells can result in chromosome mis-segregation during mitosis, leading to micronuclei generation that can also result in an accumulation of NAS in the cytoplasm and activation of the cGAS/STING pathway (Harding et al., 2017; Mackenzie et al., 2017; Li and Chen 2018). In addition, mitochondrial defects (not discussed here) can affect the CGAS/STING pathway. In summary, there exists a delicate balance between de-regulation of repeats, DDR, and the activation of innate and adaptive immune responses, all of which contribute to the intrinsic immunogenicity and fitness of the tumor.

### Lines of Defense

The regulation of genomic repeats can be altered in tumor cells when compared to differentiated somatic cells, since tumor cells undergo epigenetic re-programming and lose DNA methylation in repeat regions (Figure 3, left panel), a phenotype first described by Peter Jones and others (Jones and Taylor 1980). Due to the many detrimental effects that repeat element de-regulation can have on tumor cells, evolutionary mechanisms to counteract these adverse effects have emerged, some of which are outlined below. These can be sub-divided into epi-transcriptomic (epigenetic DNA and RNA modifications) and post-transcriptional mechanisms (including events such as RNA editing, splicing, and modification of proteins), a sub-division that is complicated by the fact that many of these factors have a multitude of reported functions.

### Epi-Transcriptomic Defense Mechanisms

Throughout evolution, primate genomes have been modified by waves of retrotransposon insertions (Cordaux and Batzer 2009; Feschotte and Gilbert 2012). Following an evolutionary period of expansion, the host species eventually managed to repress retrotransposons and prevent further insertions. In this context, it has been suggested that the expansion of the Krab Zinc Finger (KZNF) transcription factor family has contributed to the ability of primates to control the propagation of retrotransposons (Jacobs et al., 2014). The identity of specific KZNF genes that “battle” retrotransposons that are currently active in the human genome is not completely known.

Transcription of TEs is often repressed by epigenetic mechanisms such as DNA methylation and post-translational modifications (PTMs) of histone tails, some of which require KZNF for their recruitment. DNA methylation is initiated by *de novo* DNA methyl transferases such as DNMT3A/B and maintained by DNMT1. Repressive histone PTM signatures in genome repeat regions include demethylation of histone H3 Lysine 4 (H3K4), as well as methylation of histone H3 Lysine 9 (H3K9), histone H3 lysine 27 (H3K27), and histone H4 Lysine 20 (H4K20). A recent review has summarized many of the factors that can contribute to compensatory epigenetic repression of repeat regions in cancer cells, including histone methyltransferases such as Set Domain Bifurcated histone Lysine methyltransferase 1 (SETDB1), Enhancer of Zezte Homolog 2 (EZH2), and Euchromatic Histone Lysine N-methyltransferase 2 (EHMT2/G9a), histone demethylases (HDMs) such as Lysine Specific Demethylase 1 (LSD1) and Lysine Demethylase 5 (KDM5), as well as histone deacetylases (HDACs) (Chen et al., 2021). In addition to their role in promoting tumor evolution by increasing fitness and decreasing the immunogenicity of the tumor cells (Chen et al., 2021), such factors have also been shown to promote the survival of DTPs during otherwise lethal drug exposures (Guler et al., 2017; Deblois et al., 2020). Below, we will briefly outline some of the roles that such factors may play in the evolution of tumors and drug resistance in the context of genome repeat biology.

The H3K9 methyltransferase SETDB1 is over-expressed or amplified in various cancer types, correlating with poor patient survival. During development, SETDB1 has been shown to play a role in the repression of TEs and in the survival of primordial germ cells (Karimi et al., 2011). It is not fully understood to what extent SETDB1’s role in cancer development can be attributed to its ability to repress the expression of TEs, but SETDB1 loss has been shown to result in TE activation and loss of cellular fitness driven by a viral mimicry response in some cancers (Cuellar et al., 2017; Gu et al., 2021). It has also been shown that SETDB1 and increased H3K9 methylation of TEs, including evolutionarily young LINE-1 elements, play a role in the survival of DTPs following potentially lethal drug exposures (Guler et al., 2017). In addition, a recent *in vivo* IO CRISPR screen in mouse tumors identified SETDB1 as a suppressor of intrinsic tumor immunogenicity (Griffin et al., 2021). In the context of some mouse tumors, rather than unleashing an IFN response, loss of SETDB1 seemingly triggers the presentation of TE-encoded retroviral antigens leading to a TE-specific cytotoxic T cell response (Griffin et al., 2021), a mechanism that may not be conserved in all mouse tumors (Zhang et al., 2021). It is not known whether loss of SETDB1 results in the presentation of TE-derived peptides in human tumors. In these contexts, it should also be noted that repeat regions are different in mice and humans and that there may be alterations in the components of IFN signaling pathways in some inbred strains of mice (Sookdeo et al., 2013; Zhao et al., 2021). Further studies are required to clarify potential context-specific functions of SETDB1 in promoting tumor cell survival.

Members of the KDM5 family of HDMs may act as a line of defense against TE de-repression through both transcriptional and post-transcriptional mechanisms. KDM5 paralogs have been proposed to dampen viral mimicry by repressing STING expression, but it remains to be established whether it does so directly or indirectly by repressing the activation of TEs (Wu et al., 2018). A recent study suggests that KDM5B may contribute to SETDB1 recruitment, thereby leading to TE repression in the context of mouse melanoma (Zhang et al., 2021). KDM5 has also been proposed to inhibit phosphorylation and activation of TBK1 in a demethylase-dependent manner, resulting in blunted IFN signaling in breast cancer cells (Shen et al., 2021). In addition, there have been several IO-oriented CRISPR screens in mice that implicate KDM5 as having a tumor-intrinsic role in repressing IO responses (Manguso et al., 2017; Li et al., 2020). Future studies will be required to better delineate the roles that various KDM5 family members play in response to IO therapy. In the context of resistance to other drugs, there have been several reports describing a role for KDM5 family members in the establishment of DTPs (Roesch et al., 2010; Sharma et al., 2010; Vinogradova et al., 2016). However, in contrast to what is known about the role of SETDB1 in repressing LINE-1s in DTPs, it is currently not known if KDM5 functions through suppression of the viral mimicry response to promote survival of cancer cells during otherwise lethal drug exposures.

KDM5 family members and SETDB1 contribute to low H3K4 methylation and high H3K9 methylation, respectively, in the genome, and these marks serve as a recruiting platform for ATRX/DAXX and *de novo* DNA methyltransferases (DNMT) 3A, B and L (Iwase et al., 2011; Pham et al., 2020). ATRX/DAXX has been shown to contribute to the deposition of H3.3 in repetitive parts of the genome (Maze et al., 2013), and DNMT3A/B and L recruitment can result in *de novo* DNA methylation; both are mechanisms that can lead to compensatory TE repression. In this context, it is noteworthy that ATRX and H3.3, in addition to KDM5 and SETDB1, have all been shown to play a role in DTP survival (Guler et al., 2017). The chromatin remodeler ATRX has also been shown to be associated with the regulation of telomeric R-loops in ALT-dependent tumors, where it may suppress the occurrence of deleterious DNA secondary structures that form at transcribed telomeric repeats, which can induce replication fork stalling, DDR, and NAS (Abdisalaam et al., 2020). Interestingly, ATRX is often lost in ALT + tumors, and recent reports suggest that ATRX-loss is involved in ALT pathway activation by inducing telomere replication dysfunction (Haase et al., 2018; Li F. et al., 2019). It is currently unknown whether the epigenetic landscape in telomeric regions contributes to the recruitment of ATRX. It is also unknown whether ATRX, KDM5 and/or SETDB1 are involved in the suppression of R loops, rather than, or in addition to, NAS suppression in tumor cells or during the establishment of DTPs.

Another protein that may have both transcriptional and post-transcriptional regulatory roles as a defense against inappropriate TE activation is the arginine methyltransferase 5 (PRMT5). Like SETDB1, PRMT5 plays an important roler in primordial germ cell survival (Kim et al., 2014). As an epigenetic regulator, it symmetrically di-methylates arginine residues on histones H4R3, H3R8, and H2AR3. PRMT5 also interacts with Ubiquitin-like with PHD and Ring Finger Domains 1 (UHRF1), a factor that can coordinate both DNA methylation and histone modifications and has known roles in the repression of retrotransposons in the mammalian germline (Dong et al., 2019). It has also been proposed that both PRMT5 and UHRF1 can interact with P-element-induced wimpy testis (PIWI) proteins and thus regulate PIWI-interacting RNAs (piRNAs). The primary function described for piRNAs in model organisms is the silencing of retrotransposons in the germline, but piRNAs may also play a role in TE regulation in cancer (Liu et al., 2019). Non-histone substrates for PRMT5 include components of the RNA splicing machinery. Altered splicing and retention of un-spliced introns, which may contain Alu or LINE-1 element sequences, can trigger a viral mimicry response, presenting another mechanism by which PRMT5 may regulate tumor fitness and immunogenicity. PRMT5 has also been shown to regulate the ability of cGAS to bind NAS in the cytoplasm (Ma et al., 2021). Which of the many functions ascribed to PRMT5 that contributes to its tumor-promoting functions is not fully understood. Although it is possible that PRMT5 could contribute to therapy resistance and the establishment of DTPs, there are currently no studies that have described such a function.

The Methyltransferase-like 3 and 14 (METTL3/14) complex modifies RNA on adenosines at the N6 position (m6A), a dynamic epi-transcriptomic modification that has been shown to regulate critical aspects of eukaryotic RNA metabolism in numerous biological processes (Liu et al., 2020). Several roles for METTL3 in cancer and therapy responses have been described (Zeng et al., 2020). In the context of the repeat genome, it is interesting to note that the METTL3/14 complex and other factors that bind to m6A have been shown to decrease the stability of ERV-derived transcripts (Chelmicki et al., 2021). Whether m6A modifications can have effects on other NAS, and thereby affect tumor cell fitness or immunogenicity, is currently unknown. It has also recently been proposed that METTL3 can affect the ability of the RNA editing protein adenosine deaminase RNA specific 1 (ADAR1) to edit RNA by a few different mechanisms (Xiang et al., 2018; Tassinari et al., 2021). The function of ADAR in viral mimicry is further outlined below. In addition to its potential role in NAS, it has also been suggested that m6A modulation plays a role in the resolution of R-loops (Abakir et al., 2020), which could also affect DDR and viral mimicry responses in tumor settings. As mentioned above, there are many more compensatory epi-transcriptomic mechanisms that can contribute to tumor evolution or the establishment of drug resistance, and many of these are described in detail elsewhere (Chen et al., 2021).

### Post-Transcriptional Defense Mechanisms

Tumors may also employ post-transcriptional defense systems to mitigate potentially deleterious biological effects caused by TE de-regulation. Such mechanisms include modifications of signaling pathways as well as the functions of RNA-editing factors, various ribonucleases (RNases), and other nucleases and dNTPases.

The RNA editing protein family ADAR recognizes dsRNA molecules and deaminates adenosines to generate inosines (A:I editing), thereby disrupting the normal A:U pairing. The ADAR family consists of the catalytically active ADAR1 and 2 proteins, and the catalytically inactive ADAR3 protein. The most highly expressed ADAR1 protein consists of 2 isoforms: a constitutively active nuclear p110 isoform, and an IFN-inducible cytoplasmic p150 isoform (Patterson and Samuel 1995), both of which have a deaminase domain and dsRNA and Z-DNA binding domains. Loss-of-function mutations in ADAR1 have been associated with interferonopathies such as Aicardi-Goutieres syndrome (AGS) (Crow and Stetson 2021). In the context of cancer, ADAR1 has been shown to decrease the viral mimicry response associated with dsRNA recognition of inverted Alu repeats by MDA5 (Mehdipour et al., 2020). Given that the p150 isoform of ADAR1 is activated by IFN signaling, it can act as a feedback mechanism in the viral mimicry response induced by Alu repeats. It has also been shown that ADAR1 loss of function in tumor cell models sensitizes them to immunotherapy and overcomes resistance to checkpoint blockade (Ishizuka et al., 2019). Taken together, these observations suggest that ADAR1 helps cancer cells to suppress the inflammatory response driven by TE activation, thereby avoiding immunosurveillance or reduced fitness that could be driven by translational changes linked to IFN-induced RNAse L and PKR activities (Lamers et al., 2019). The constitutively expressed p110 ADAR isoform has been shown to suppress telomeric R-loops due to its ability to edit mismatched telomeric repeats (Shiromoto et al., 2021). Although there is extensive literature that supports a role for ADAR1 in decreasing IO responses (Ishizuka et al., 2019; Mehdipour et al., 2020), there is no current literature that describes a role for ADAR1 in affecting other drug responses. However, a recent report has proposed that loss of ADAR2 can render tumor cells hypersensitive to genotoxic agents, dependent on the ability of ADAR2 to edit DNA/RNA hybrids during damage (Jimeno et al., 2021).

The three-prime repair exonuclease 1 (TREX1) enzyme is a 3′-5′ DNA exonuclease that can target reverse-transcribed TE-derived cDNAs and prevent their cytosolic accumulation (Stetson et al., 2008; Li et al., 2017). TREX1 can act on both ssDNA and dsDNA (Mazur and Perrino 1999). Like ADAR, mutations in TREX1 are associated with autoimmune diseases such as AGS (Crow and Stetson 2021). It has been shown that DNA-damaging agents used in chemotherapy can result in ssDNA release into the cytosol, resulting in enhanced activation of the cGAS/STING pathway in the absence of TREX1 (Erdal et al., 2017). Similarly, TREX1 can degrade DNA derived from micronuclei in chromosomally unstable tumor cells and prevent activation of cGAS/STING (Mohr et al., 2021). In addition to restricting NAS, TREX1 may also dampen the activation of the DNA damage checkpoint by preventing S-phase accumulation of ssDNA (Yang et al., 2007). Therefore, like ADAR1, TREX1 may inhibit both activation of NAS and DDR pathways in the context of cancer and its activities could affect drug responses.

The activated-induced cytidine deaminase (AID) and Apolipoprotein B mRNA editing enzyme, catalytic polypeptide-like (APOBEC) proteins deaminate cytidine residues in DNA and RNA and can therefore affect a wide range of cellular functions, including restriction of exogenous and endogenous viruses (Esnault et al., 2005; Harris and Dudley 2015). AID was originally identified as an enzyme that plays a role in somatic hypermutation and class switch recombination, serving to generate mutations that diversify immunoglobulin genes (Muramatsu et al., 2000). It has since been shown to also restrict viral replication, along with APOBECs (Gourzi et al., 2006; Malim 2009). The best studied human APOBEC proteins belong to the APOBEC3 subfamily, and all members (A3A-D, A3F-H) have been reported to inhibit LINE-1 retrotransposition, as well as exogenous viral replication, through a still undefined mechanism (Kinomoto et al., 2007; Harris and Dudley 2015). The ssDNA component of R-loops is also prone to cytidine deamination by AID/APOBEC family members, resulting in CAG repeat breaks and DDR (Su and Freudenreich 2017). Interestingly, AID/APOBEC family members have another ascribed function due to their ability to deaminate 5-methylcytosine (5mC) to a thymine (T), resulting in T:G mismatches and DNA demethylation following repair (Morgan et al., 2004). There have also been described roles in tumors for AID/APOBEC proteins in producing genome-wide mutations and DSBs that can result in tumor-promoting DNA translocations (Okazaki et al., 2003; Robbiani et al., 2008; Robbiani et al., 2009) Through their effect on the mutational landscape and the ability to restrict endogenous retrovirus replication, it is possible that AID/APOBEC proteins can affect therapy resistance. Interestingly, it has been shown that ABOBEC3 RNA levels are induced in some DTPs (Guler et al., 2017), but it is currently unknown whether APOBECs or other editing enzymes contribute to mutational drug resistance or DTP survival due to its ability to perturb viral mimicry.

The ATP-dependent RNA helicase Moloney leukemia virus 10 (MOV10) was first identified as a protein that inhibits infection of Moloney leukemia virus in mice (Jaenisch et al., 1981; Mooslehner et al., 1991). In cell culture, MOV10 has been shown to bind LINE-1 transcripts and ribonucleoproteins and inhibit retrotransposition of TEs (Arjan-Odedra et al., 2012; Goodier et al., 2012; Lu et al., 2012). While the exact mechanism of MOV10-mediated restriction remains unclear, studies have shown that MOV10 promotes nonsense-mediated mRNA decay, as MOV10 knockdown increased half-lives of MOV10-bound transcripts (Gregersen et al., 2014). It has been proposed that MOV10 may unwind mRNA secondary structure and displace proteins at the 3′UTR that protect them from decay. While evidence of the role of MOV10 in cancer and/or the viral mimicry response is scant, some studies have shown that MOV10 participates in tumor emergence and progression (Nakano et al., 2009; El Messaoudi-Aubert et al., 2010; Yang et al., 2019; Mao et al., 2020). It has also been suggested that MOV10 can bind RNase H2 and/or Zinc-finger antiviral protein (ZAP) to prevent DNA/RNA hybrid formation during L1 retrotransposition, which may affect NAS/DDR in some contexts (Moldovan and Moran 2015). The functional roles of RNASE H1 and 2 will be described below. ZAP is a member of the poly (ADP-ribose) polymerase (PARP) family, which binds to repetitive RNA sequences leading to their degradation. So far, in humans, four alternatively spliced ZAP isoforms have been identified, consisting of short and long isoforms (ZAP-S and ZAP-L respectively) (Kerns et al., 2008; Li M. M. H. et al., 2019). Though these isoforms are similar, it has been suggested that ZAP-S is associated with the viral sensor RIG-I and a type I IFN response (Hayakawa et al., 2011). ZAP can inhibit both human LINE-1 and Alu retrotransposition in tissue culture models, and many cancer contexts seem to “prefer” low ZAP levels (Liu Y. et al., 2018; Cai et al., 2020). However, specific roles for ZAP and MOV10 restriction factors in cancer development and drug resistance have yet to be defined.

Several RNases have been shown to protect against the potentially deleterious effects of de-repression of TEs or de-stabilization of telomeric sequences. These include Ribonuclease L 2′, 5′-oligoisoadenylate synthetase-dependent ribonuclease (RNase L), RNase H1 and RNase H2. RNase L is an IFN-inducible endoribonuclease that binds and cleaves single-stranded RNA molecules (Goodier 2016). The dimerization and activation of RNase L are driven by NAS-induced expression of oligoadenylate synthetase (OAS) (Silverman 2007), leading to translational arrest, autophagy and/or apoptosis. RNase L over-expression has been shown to restrict LINE-1 and ERV activities in cultured human cells (Zhang et al., 2014). Other RNases whose functions can affect NAS and/or DDR responses include RNase H1 and RNase H2 enzymes. The RNase H family of proteins can suppress the accumulation of R-loops through the endonucleolytic cleavage of RNA in RNA/DNA hybrids (Wang et al., 2018). It should also be noted that RNase H family members may function in restricting other DNA/RNA hybrids such as reverse transcribed sequences (Zhao et al., 2021). RNase H1 functions as a monomer independently of the cell cycle, whereas RNase H2 is comprised of 3 subunits that are expressed in a cell cycle-dependent manner (Lockhart et al., 2019). Like mutations in many other enzymes that control NAS, including ADAR1 and TREX, as well as SAM and HD domain containing Deoxynucleoside Triphosphate Triphosphohydrolase1 (SAMHD1, below), mutation of any of the three RNase H2 subunits has been demonstrated to contribute to AGS (Crow et al., 2006; Crow and Stetson 2021). Several reports have also suggested that both RNase H1 and RNase H2 activity can contribute to an effective DDR (Amon and Koshland 2016). For example, RNase H2 is involved in Ribonucleotide Excision Repair (RER), in a process where mis-incorporated ribonucleotides are excised from duplex DNA. RNase H1 can also regulate the levels of DNA/RNA hybrids at telomeric repeats and is a key mediator of telomere maintenance in ALT-dependent tumors (Arora et al., 2014). As of yet, no clear roles for the RNase H family of enzymes in the development of tumors or drug resistance have been reported, but their functions highlight possible mechanisms for crosstalk between de-regulation of the repeat genome, its restriction factors, and NAS/DDR. Consistent with these observations, loss-of-function mutations in the RNase H2 enzyme limit the processing of DNA/RNAhybrids and genome mis-incorporated ribonucleotides, leading to increased cGAS/STING activation (Mackenzie et al., 2016). This suggests that loss of RNase H2 in tumors with high TE expression or a high content of endogenous R-loops could stimulate an IFN response.

Other defense factors that may function in both DDR and NAS include: 1) SAMHD1 and 2) cGAS, both of which have cytoplasmic and nuclear functions, and 3) primase/polymerase (PrimPol). SAMHD1 was initially identified as a cellular restriction factor for Human Immuno-Deficiency Virus-1 (HIV-1) in nondividing myeloid cells (Hrecka et al., 2011; Laguette et al., 2011). Early studies defined a role for SAMHD1 in regulating dNTP pools in proliferating mammalian cells (Franzolin et al., 2013), but more recent findings have revealed that SAMHD1 may also suppress innate immune responses to viral infection (Chen et al., 2018). In addition to its ability to restrict exogenous viruses, SAMHD1 has been shown to restrict non-LTR retrotransposons, a function that is not completely understood and may be independent of its dNTPase activity (Zhao et al., 2013). In the context of DDR, SAMHD1 can promote the degradation of the nascent DNA strand at stalled replication forks by stimulating the exonuclease activity of MRE11 (Coquel et al., 2018). This creates exposed ssDNA, which activates the Ataxia Telangiectasia and Rad3-related protein (ATR) checkpoint, promoting replication fork restart. Therefore, loss of SAMHD1 in tumor cells could have several consequences, including loss of checkpoint response, increase in cytoplasmic NAS derived from TEs, and increase in ssDNA fragments in the cytosol, released from stalled replication forks. These findings suggest that SAMHD1 may contribute to the suppression of both DDR and NAS in tumor cells. Although some tumors show sensitivity to SAMHD1 loss, future experiments are needed to determine whether SAMHD1 plays a general role in tumorigenesis and drug resistance.

As discussed above, the cGAS–STING pathway is an essential component of the innate immune system that functions to detect the presence of cytosolic DNA or DNA/RNA hybrids (Ishikawa et al., 2009; Barber 2015). In addition to its canonical role in sensing NAS in the cytoplasm, cGAS has been shown to interact with replication fork proteins and act as a decelerator of DNA replication forks. As a result, cGAS deficiency in tumors can lead to compromised replication fork stability and increased sensitivity to radiation and chemotherapy (Chen et al., 2020). It has also been suggested that micronuclei arising from genome instability and chromosome mis-segregation can lead to accumulation and activation of cGAS, providing a cell-cycle-dependent mechanism by which cGAS can detect self-DNA (Mackenzie et al., 2017). Other studies have reported an opposite view where nuclear cGAS suppresses homologous recombination (HR) and DNA repair (Liu H. et al., 2018; Jiang et al., 2019). While one study suggests that cGAS-mediated inhibition of HR repair promotes genomic instability and tumorigenesis in a mouse model of lung cancer (Liu H. et al., 2018), another study reported that inhibition of HR repair by cGAS in mouse bone marrow-differentiating monocytes promotes irradiation-induced cell death (Jiang et al., 2019). This suggests that cGAS inhibition in the presence of DNA-damaging agents may result in reduced cell death in normal cells while sensitizing tumor cells to such therapy. However, it should be noted that radiation and chemotherapy can also induce NAS, and cGAS deficiency or inhibition may reduce the activation of IFN signaling in this context. Taken together, these studies paint a picture wherein cGAS may have functions unrelated to its role in STING signaling, and future studies will be needed to more clearly establish the roles that cGAS plays in tumor development and drug responses.

Another protein sharing the intricate duality as a DDR and NAS factor is PrimPol (Mouron et al., 2013). This enzyme can prime DNA synthesis using template pyrimidines (Bianchi et al., 2013; Garcia-Gomez et al., 2013), but also exhibits DNA polymerase activity, capable of extending DNA/RNA chains. Using its primase activity, PrimPol can re-prime stalled replication forks, generating ssDNA gaps (Lopes et al., 2006; Bai et al., 2020; Quinet et al., 2020; Genois et al., 2021). In the context of repeated sequences, the absence of PrimPol has been shown to result in increased R-loop formation (Svikovic et al., 2019), potentially releasing DNA/RNA hybrids into the cytosol where they can activate an innate immune response via the cGAS/STING pathway. Similarly, the Fanconi anemia protein FANCM is involved in disrupting TERRA R-loops at telomeric regions in ALT-dependent tumors (Pan et al., 2019). This mechanism prevents the replisome from stalling and potentially precludes activation of the innate immune response. Another DDR factor, the DNA-dependent protein kinase complex (DNA-PK), involved mainly in non-homologous end-joining (NHEJ) of DSBs, has also been shown to affect IFN signaling by acting as a DNA sensor, resulting in IRF-3 activation in human and mouse fibroblasts (Ferguson et al., 2012; Burleigh et al., 2020). A similar role for DNA-PK in tumor cells has not been described. Other reports have suggested that DNA-PK phosphorylates cGAS and blocks downstream signaling (Sun et al., 2020). These are just a few examples where DNA repair proteins may affect both viral mimicry and DDR.

**FIGURE 4:**
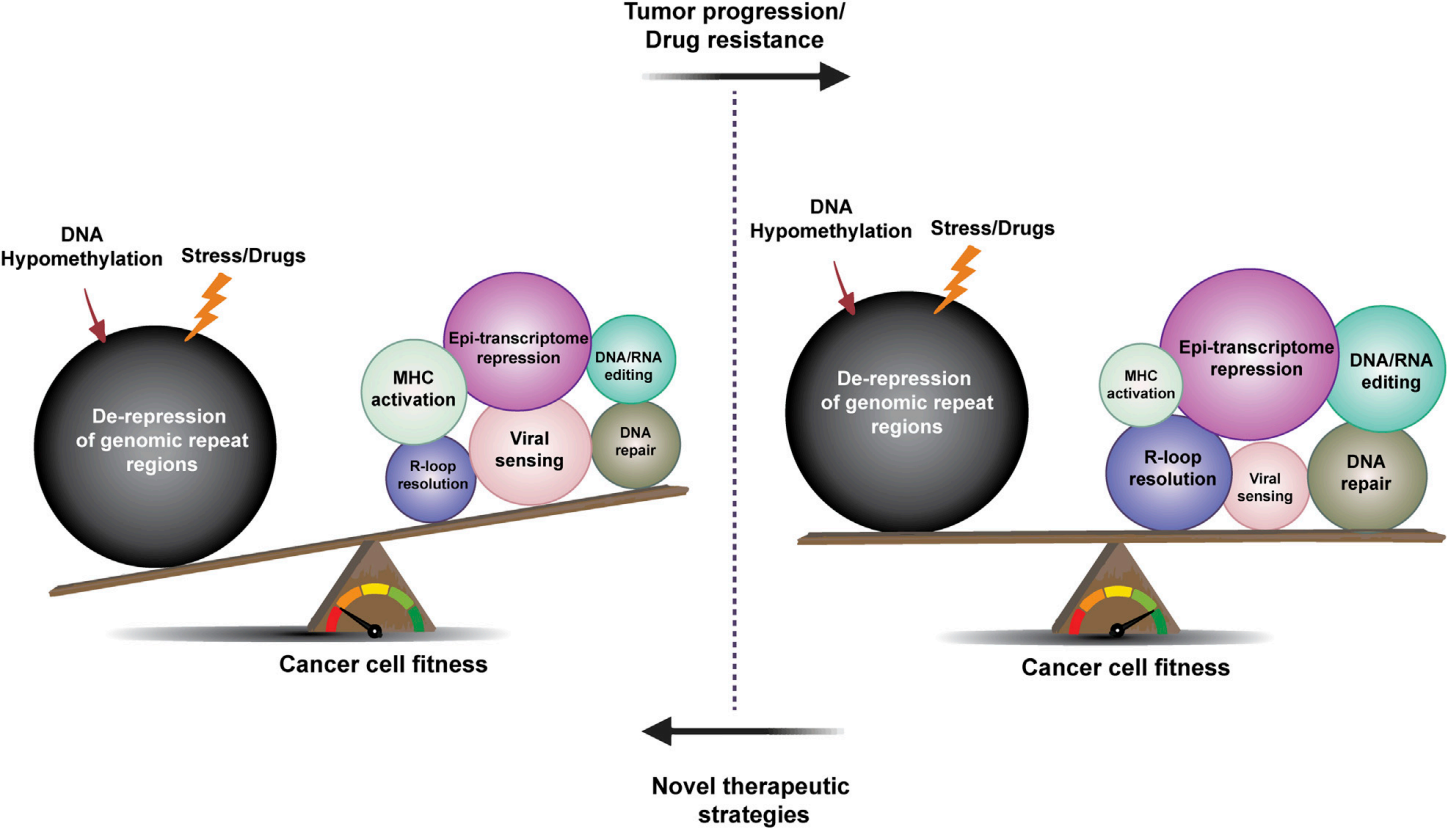
The rheostat of repeat element de-regulation and restriction in tumor evolution and drug resistance. The evolution of cancer and drug resistance may be described as a battle between factors that decrease and promote tumor fitness and immunogenicity. Upon drug-induced stress or DNA hypomethylation, de-repression of repeat elements leads to loss of cancer cell fitness and activation of innate and adaptive tumor immunity, in part driven by viral sensing and DDR mechanisms (left). Compensatory mechanisms, such as epi-transcriptome regulation, DNA repair, DNA/RNA editing, and R-loop resolution factors can all contribute to the establishment of a balance that promotes tumor development and the evolution of drug resistance (as indicated by the changes in circle size in the right part of the figure). Future studies will determine whether new tumor therapy strategies aimed at disrupting this balance can result in more curative outcomes.

In summary, the counter-balancing factors associated with curbing the consequences of TE de-regulation described above are examples from a growing body of literature that suggests extensive mechanistic crosstalk between DNA repair processes and nucleic acid-associated inflammatory responses. Indeed, factors involved in endogenous or exogenous nucleic acid-sensing or processing appear to play a dual role in the initiation of inflammatory responses on one hand, and surveillance of genomic integrity and/or DNA repair on the other hand. This suggests the existence of complex intertwined signaling networks that can, depending on the circumstances, affect genome stability and/or the immune response. It is paramount to consider these complex biological balances to better design and predict therapeutic responses and overcome or prevent drug resistance (Figures 3, 4).

## The Rheostat of Repeat Element De-Regulation and Restriction in Tumor Evolution and Drug Resistance

The notion that viruses or other microbes play a role in cancer development has been considered for more than a century. Following the discovery by Peyton Rous that the Rous sarcoma virus (RSV) can cause tumors in chickens, cancer was, for a time, considered to be a viral disease (Rous 1910). Subsequent studies have shown that other viruses, including Epstein Barr Virus (EBV) and human papillomavirus (HPV), can contribute to cancer development in humans. More recently, it has also been suggested that de-regulation of the repetitive parts of the human genome that contain endogenous viruses plays a role in tumor evolution as well as in cancer therapy resistance. Human tumor cells have been shown to harbor *de novo* insertions of TEs (Payer and Burns 2019), the majority of which may be viewed as passenger events, but some may also promote the expression of cancer-causing genes. In addition to these insertional events, de-regulation of TEs resulting from alterations in cancer cells, including DNA hypomethylation of repeat regions, can have tumor-suppressive consequences by affecting fitness as well as immunogenicity of the tumor. Therefore, tumor evolution may involve a multitude of mechanisms that create a TE “rheostat” (Figure 4) that can promote tumor survival--reminiscent of the toxin/antitoxin systems and other antibiotic resistance mechanisms in bacteria (Fasani and Savageau 2013; Ghosh et al., 2020).

A few studies have suggested that compensatory epigenetic switching may occur in cancer evolution following the loss of DNA methylation in repeat regions. For example, studies in AML have shown that repression of LINE-1 elements by complexes that mediate H3K9 methylation is important for AML progression, and consequently, patients whose cancer exhibited low expression of LINE-1 elements had a worse prognosis (Gu et al., 2021). A recent report has similarly suggested that de-regulated expression of genome repeats can have a tumor-suppressive effect following chemical induction of tumors in mice and blind mole rats (Zhao et al., 2021). Future studies will be required to establish the importance of the TE rheostat in tumor evolution.

In addition to a potential role for this balancing act in tumor evolution, recent reports have suggested that de-regulation of TEs may contribute to anti-cancer drug responses (innate or acquired resistance). In the context of IO, a study by Nir Hacohen and others first showed that tumors that either harbor exogenous viruses, such as EBV and HPV, or display an increase in the expression of endogenous viruses show an increase in infiltrating immune cells, suggesting that such tumors may respond better to IO therapy (Rooney et al., 2015). These observations were followed by reports showing that increased expression of endogenous viruses can create NAS in the cytoplasm of tumor cells and elicit a tumor-intrinsic IFN response and activate innate immunity, which can affect both fitness and the immunogenicity of tumor cells (Roulois et al., 2015; Chiappinelli et al., 2016). More recent studies have also suggested that de-regulated TE expression can increase neoantigen presentation, thereby potentiating the immunogenicity of tumor cells (Kong et al., 2019; Griffin et al., 2021). The initial reports of viral mimicry described the use of DNA hypomethylating agents to further de-repress TEs in tumor cells, but many subsequent studies have implicated other epi-transcriptomic factors in compensatory repression of TEs and IFN response in tumors, a mechanism that has been described as epigenetic switching (Chen et al., 2021). As outlined above, the intrinsic decrease in tumor cell fitness and/or increase in immunogenicity, associated with TE activation, can also be regulated by a myriad of other factors. For example, mutations or alterations in the viral sensing signaling pathways can compensate for TE de-regulation, and it is interesting to note that patients who relapse on IO therapy often harbor mutations in these pathways. Although the original studies of viral mimicry were focused on dsRNA species as the initiators, later studies have also implicated other NAS as inducers of innate immune responses in tumors. These species include RNA/DNA hybrids generated by reverse transcriptase or derived from R-loops, damaged DNA that can result in micronuclei formation, or leakage of mitochondrial DNAs.

Studies in colorectal tumor cells have also demonstrated that DNA hypomethylating agents negatively affect their tumor re-initiating potential (Roulois et al., 2015) - a phenotype associated with the “cancer stem cell” paradigm. In this context, we note that leukemic stem cells, observed to display decreased sensitivity to drugs and therefore might serve as reservoirs of relapse, exhibit transcriptional repression of TEs and IFN–induced pathways as compared to other leukemic cells (Colombo et al., 2017). Exposure to cancer drugs can also induce TE expression, and it has been shown that the survival of DTPs, which show some stem cell characteristics, is dependent on TE repression (Guler et al., 2017; Deblois et al., 2020). The decreased number of DTPs seen following epigenetic therapy may be due to a combination of increased genome instability and the induction of viral mimicry caused by the de-regulation of TEs in this therapy-resistant subpopulation of cells. These studies suggest that, in addition to DNA hypomethylating agents and HDAC inhibitors, tumor-specific TE repression mechanisms not used in adult somatic cells (and perhaps “borrowed” from developmental biology), could be harnessed for future drug development aimed at potentiating existing therapies, including chemotherapy and targeted agents.

In evolutionary biology, one can describe an “arms race” as an ongoing “competition” between two or more co-evolving species, genes, or traits that drive mutual adaptation or opposition. In this review, we have considered the most recent advances in the arms race between TEs and their restriction factors in the development and treatment of cancer. Since de-regulation of this balance could also contribute to the development of other diseases, it is important to consider that cancer cells are characterized by a multitude of changes that may be unique, including changes to the epigenome, which might generate a tumor-specific “Achilles’ heel” that can be exploited with drug treatment. In closing, many studies have significatively contributed to our understanding of the de-regulation of TE biology in cancer development and therapy response, but there are still unanswered questions that will require future studies to address. We must also consider that 1) most human tumor studies have so far focused on the “fitness” of the tumor and not necessarily on tumor cell-intrinsic changes that affect immune responses, and 2) the fact that mouse and human repeat regions differ, which may have to be considered as we translate studies in mice to human cancer biology. Future studies will have to evaluate how different tumors establish the balance that counteracts the deleterious effects of TE de-regulation to better understand how to exploit these mechanisms to achieve longer-term cancer remissions and cures (Figure 4).

## Data Availability

The original contributions presented in the study are included in the article/Supplementary Material, further inquiries can be directed to the corresponding authors.
